# Tanshinone IIA Inhibits Growth of Keratinocytes through Cell Cycle Arrest and Apoptosis: Underlying Treatment Mechanism of Psoriasis

**DOI:** 10.1155/2012/927658

**Published:** 2011-11-16

**Authors:** Fu-Lun Li, Rong Xu, Qing-chun Zeng, Xin Li, Jie Chen, Yi-Fei Wang, Bin Fan, Lin Geng, Bin Li

**Affiliations:** ^1^Department of Dermatology, Yueyang Hospital of Integrated Traditional Chinese and Western Medicine, Shanghai University of Traditional Chinese Medicine, Shanghai 200437, China; ^2^Department of Critical Care Medicine, Affiliated Hospital of Guangdong Medical College, Zhanjiang, Guangdong 524001, China

## Abstract

The aim of the present investigation was to elucidate the cellular mechanisms whereby Tanshinone IIA (Tan IIA) leads to cell cycle arrest and apoptosis *in vitro* in keratinocytes, the target cells in psoriasis. Tan IIA inhibited proliferation of mouse keratinocytes in a dose- and time-dependent manner and induced apoptosis, resulting in S phase arrest accompanied by down-regulation of pCdk2 and cyclin A protein expression. Furthermore, Tan IIA-induced apoptosis and mitochondrial membrane potential changes were also further demonstrated by DNA fragmentation, single-cell gel electrophoresis assay (SCGE), and flow cytometry methods. Apoptosis was partially blocked by the caspase-3 inhibitor Ac-DEVD-CHO. Mitochondrial regulation of apoptosis further downstream was investigated, showing changes in the mitochondrial membrane potential, cytochrome c release into the cytoplasm, and enhanced activation of cleaved caspase-3 and Poly ADP-ribose polymerase (PARP). There was also no translocation of apoptosis-inducing factor (AIF) from mitochondria to the nucleus in apoptotic keratinocytes, indicating Tan IIA-induced apoptosis occurs mainly through the caspase pathway. Our findings provide the molecular mechanisms by which Tan IIA can be used to treat psoriasis and support the traditional use of *Salvia miltiorrhiza Bungee (Labiatae)* for psoriasis and related skin diseases.

## 1. Introduction

**Figure 1:**
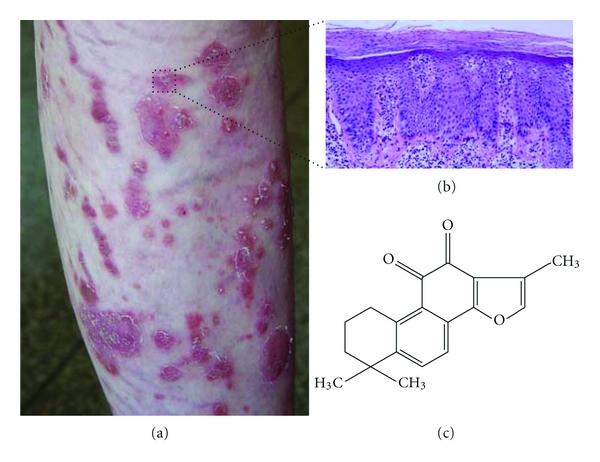
Clinical and pathological presentation of psoriasis and chemical structure of tanshinone IIA. (a) A 55-year-old woman with psoriasis suffered from erythematous plaques with limited silvery sale on her legs lasting as long as 5 years. (b) Epidermal regular psoriasiform hyperplasia in the epidermis and parakeratosis with Munro's microabscesses. (c) Chemical structure of tanshinone IIA.

Psoriasis is a chronic inflammatory skin disease affecting 0.6% to 4.8% of people worldwide [1]. Excessive proliferation of keratinocytes, abnormalities in the differentiation process, and continuous shedding of the thickened epidermis are characteristics of psoriasis (Figures 1(a) and 1(b)) [2]. The underlying cause of psoriasis, however, is not well understood. 

In China, patients with psoriasis often turn to alternative and complementary treatments, which are considered effective and safe [3]. The use of herbal medicines to prevent the development as well as recurrence of psoriasis and other inflammatory diseases has become widely accepted. Danshen, a traditional Chinese crude drug, was one of the most widely used in traditional Chinese medicine. Tanshinone IIA (Tan IIA) is one of the main natural active ingredients purified from *Salvia miltiorrhiza radix*, which has been widely used in clinical practice for the prevention of psoriasis, atopic dermatitis, and other inflammation-related disorders.

Other investigators have explored the involvement of Tan IIA in causing apoptosis [4–7]. Despite the tremendous progress that has been made in the therapeutic use of Tan IIA, the molecular mechanism(s) involved in the treatment of psoriasis remain(s) unresolved. Numerous studies have documented the consequences of Tan IIA treatment, including apoptotic effects against leukemia [8, 9], hepatocarcinoma [10], breast cancer [11], colon cancer [11], and gliomas [12]. Although, most of these studies have focused on treatment of tumors, the effects and mechanism of action in keratinocytes are still poorly understood. Therefore, based on clinical experience as well as cytotoxic activity against multiple human cancer cells, we hypothesized that induction of apoptosis is the underlying mechanism for the treatment of psoriasis. We investigated the effects of various concentrations of Tan IIA (5–10 *μ*g/mL) on mouse keratinocytes and human HaCat cells *in vitro* to confirm this hypothesis. Protein and gene expression of apoptotic signaling pathway-related proteins such as caspase-3, cytochrome c, and PARP were determined to provide evidence for the mechanisms of action of Tan IIA in the treatment of psoriasis.

## 2. Materials and Methods

### 2.1. Chemicals and Reagents

Tan IIA (Figure 1(c); 99.2% purity) was purchased from the National Institute for the Control of Pharmaceutical and Biological Products (Shanghai, China) and completely dissolved in DMSO (shake overnight in 37°C) at a stock concentration of 4 mg/mL. Propidium iodide (PI) and the enhanced apoptotic DNA ladder detection kit were purchased from BioVision Inc. (Mountain View, CA, USA). Apo-BrdU apoptosis detection kit and mitochondrial membrane potential detection kit were purchased from BD Biosciences (San Diego, CA, USA). An OxiSelect Comet Assay Kit was purchased from Cell Biolabs Inc. (San Diego, CA, USA). Antibodies for detection of the apoptosis and cell cycle-related proteins caspase-3 (1 : 200), PARP (1 : 1000), PCNA (1 : 500), AIF (1 : 500), and pCDK2 (1 : 200) were purchased from Cell Signaling Technology (Beverly, MA, USA). Cyclin A and cytochrome c were purchased from BD Biosciences (San Diego, CA, USA) and *α*-tubulin was purchased from Sigma Chemical Co. (St. Louis, MO, USA). ELISA testing kits for caspase-3 and -8 were purchased from BioVision Company (Mountain View, CA, USA).

### 2.2. Cell Culture

Primary mouse keratinocytes were prepared from newborn mice as previously described [13], and cultured in Cnt-07 medium and supplements (Cellntec, Berne, Switzerland). Dr. Juan Wang at the Shanghai Institute of Pharmaceutical Industry kindly provided the HaCat human keratinocyte cell line. HaCat cells were cultured in Dulbecco's modified Eagle's medium (DMEM; Invitrogen, Carlsbad, CA, USA.) supplemented with 10% heat-inactivated fetal bovine serum (FBS) and 100 U/mL penicillin. The cells were incubated at 37°C in an atmosphere of 5% CO_2_.

### 2.3. Cell Growth Inhibitory Rate (MTS/PMS) Assay

Cell growth inhibitory rate was assayed using the MTS/PMS method. Briefly, cells (1 × 10^3^/well) were plated in 96-well culture plates (Nunclon, Nunc, Roskide, Denmark). After incubation overnight, different concentrations of Tan IIA (2.5, 5, or 10 *μ*g/mL) were added to various wells. Each of the concentrations above was regarded as one treated group while the control group only contained DMSO. Each group contained 6 replicate wells. After culture plates were incubated for 0, 24, 48, or 72 h, 100 *μ*L of MTS/PMS working solution was added to each well and then the keratinocytes were incubated continuously for another 4 h. The absorbance (A value) of each well was measured using a spectrophotometer (Beckman Coulter; Miami, FL, USA) at 490 nm. The effect of Tan IIA on the cell growth inhibitory rate in keratinocytes was calculated according to the following formula: % inhibitory rate = A_490_ value of treated group/A_490_ value of control group ×100%. The 50% inhibitory concentration (IC_50_) was determined from dose-response data from at least 3 independent experiments.

### 2.4. Clonogenic Survival Determination

Cells were assayed for their colony-forming ability by replating them at specified numbers (300–400 cells/well) in 6-well plates treated with 2.5, 5.0, or 10.0 *μ*g/mL of Tan IIA. After 7 days of incubation, cells were stained with 0.5% crystal violet in absolute ethanol, and colonies with >50 cells were counted under a dissecting microscope.

### 2.5. Cell Cycle Analysis

Cells were plated at a density of 1.5 × 10^6^ per 6 cm diameter dish and allowed to grow for 24 h, after which the medium was changed to serum-free medium. After 16–18 h of starvation, cells are synchronized in the G_0_ phase of the cell cycle. Different experimental reagents were then added to the serum-free medium. Cells (1 × 10^6^) were harvested by trypsinization after the indicated treatment, washed twice in cold PBS, and then fixed in 70% alcohol for 30 min on ice. After washing in cold PBS three times, cells were resuspended in 1 mL of Krishan staining solution [14] overnight at 4°C. A 96-micron pore size nylon mesh was used to filter cells the next day, and a total of 10,000 stained nuclei were analyzed with a flow cytometer (BD Biosciences; San Jose, CA, USA). DNA histograms were prepared using the ModFit analysis program (BD Biosciences; San Jose, CA, USA), which fits the fractions of cells in the G_0_-G_1_, S, and G_2_-M phases of the cell cycle. Each condition was repeated in triplicate.

### 2.6. Flow Cytometry Analysis for Cell Apoptosis

Mouse keratinocytes in log-phase growth were exposed to different concentrations of Tan IIA (from 2.5 to 10.0 *μ*g/mL), harvested, and processed for flow cytometry (Beckman Coulter Gallios) using the TUNEL-based Apo-BrdU Kit (BD Biosciences), according to the manufacturer's instructions. Floating and attached cells were incubated with DNA labeling solution containing BrdU, harvested at 24, 48, and 72 h of Tan IIA exposure, and subsequently fixed with 4% paraformaldehyde. After washing, samples were incubated with fluorescein-labeled anti-BrdU, pelleted, and washed with wash buffer. Cells were incubated with RNase/PI solution provided by the Apo-BrdU Kit and kept in the dark at 4°C overnight. Cells were analyzed by flow cytometry using the doublet discrimination module and data were acquired using Cell Quest (BD Biosciences) software. The cell cycle was modeled and data were expressed as a percentage of apoptotic cells in the total cell preparation. All data were collected and analyzed by Lysis II software (Becton Dickinson). The experiments were repeated 3 times and the results are presented as mean ± SD.

### 2.7. Immunofluorescence Staining

Cells were cultured on 8-well chamber slides (Fisher Brand) and treated with different concentrations of Tan IIA for 24 h. Cells were then fixed with 4% paraformaldehyde, permeabilized with 0.3% Triton X-100, digested in pepsin solution (Lab Vision, Thermo Scientific Corporation; Waltham, MA, USA), blocked for 1 h with 10% normal goat serum plus 0.1% NP-40, and incubated with target primary antibodies overnight. Alexa Fluor 488- or 594-conjugated secondary antibodies (Invitrogen) were used for immunofluorescence staining, and images were taken using confocal microscopy with a Leica SP2 confocal microscope (Leica Microsystems Inc.; Deerfield, IL, USA).

### 2.8. DNA Fragmentation Assay

Apoptosis was determined with the enhanced apoptotic DNA ladder detection kit according to the manufacturer's instructions (BioVision Company). Briefly, keratinocytes (10 × 10^5^) were collected, washed with PBS twice, and cells were pelleted by centrifugation for 5 min at 500 ×g. Enzyme B (protein kinase) solution (5 *μ*L) was added to each sample and they were incubated at 50°C overnight. Cells were then lysed with 35 *μ*L of TE lysis buffer (50 mM Tris-HCl [pH 7.4], 20 mM EDTA containing 1% sodium dodecyl sulfate [SDS]), and incubated at 37°C for 10 min. Ammonium acetate solution (5 *μ*L) was added to each sample, mixed well, 50 *μ*L of isopropanol was added, and samples kept at −20°C for 10 min. Samples were then centrifuged for 10 min to precipitate DNA, the pellet was washed with 0.5 mL of 70% ethanol, and air dried for 10 min at room temperature. The DNA pellet was dissolved in 20 *μ*L of DNA suspension buffer, electrophoresed on a 1.8% agarose gel, visualized under UV light, and photographed.

### 2.9. Single-Cell Gel Electrophoresis Assay

The single-cell electrophoresis assay was performed using the Comet Assay Kit (Cell Biolabs Inc., San Diego, CA, USA). Briefly, keratinocytes were treated with 10 *μ*g/mL of Tan IIA for 24 h. The cells were then washed with cold PBS, scraped, collected into 1.5 mL tubes, and centrifuged. The cell pellet was then combined with Comet Agarose at 1 : 10 ratio (v/v), mixed, and 75 *μ*L/well was immediately pipetted onto the Comet assay slide. The slides were dried completely at 4°C in the dark for 15 min followed by incubation in prechilled Lysis solution at 4°C in the dark for another 15 min. After washing with TBE buffer (50 mM Tris, 50 mM boric acid, and 0.2 mM EDTA), the samples were subjected to electrophoresis at 31 V for 30 min in TBE and stained with vista green DNA dye. The signal was detected with a Nikon fluorescent microscope (Nikon; Tokyo, Japan). Tail DNA% of 100 cells in each slide was measured using NIH Image J software (http://rsb.info.nih.gov/ij/; NIH; Bethesda, MD, USA).

### 2.10. Western Blot Analysis

Western blot analysis was completed as described by Sambrook et al. [15]. Keratinocytes were cultured as before. After culture, the cells were harvested and lysed with lysis buffer (20 mM Tris-HCl (pH 7.5), 150 mM NaCl, 1 mM Na_2_EDTA, 1 mM EGTA, 1% Triton, 1%NP-40, 2.5 mM sodium pyrophosphate, 1 mM *β*-glycerophosphate, 1 mM leupeptin, and 1 mM phenylmethylsulfonyl fluoride (PMSF)), for 30 min at 4°C. The insoluble material was removed by centrifugation at 10.000 rpm for 10 min at 4°C. The concentration of protein in each cell lysate was measured with bovine serum albumin (BSA) as a protein standard (Sigma). An identical amount of protein (50 *μ*g) from each sample was loaded onto a 10% SDS-PAGE gel and electrophoresed at 100 V. Proteins were then transferred to nitrocellulose membranes (0.45 *μ*m; Millipore; Billerica, MA, USA) and blocked with 5% nonfat milk in TBS (25 mM Tris-HCl, 150 mM sodium chloride, pH 7.2) for 1 h at room temperature. The blots were incubated with primary antibodies at a dilution of 1 : 1000 in blocking buffer overnight at 4°C. The membranes were washed three times and then incubated with horseradish peroxidase- (HRP-) conjugated goat anti-rabbit/(mouse) IgG (1 : 5000 in Odyssey blocking buffer; Li-Cor Biosciences; Lincoln, NE, USA) for 1 h at room temperature. Gray-scale images were obtained and quantified with Odyssey v.1.2 software (LI-Cor Biosciences; Lincoln, NE, USA).

### 2.11. Mitochondrial Membrane Potential Assay

Cells were seeded in 12-well plates for 24 h and stained with the JC-1 reagent from BD Biosciences (San Jose, CA, USA) according to the manufacturer's instructions. After incubation for 15 min, JC-1 aggregates in the mitochondria (only in live cells) were detected as a red color by a fluorescence microscope using excitation/emission = 540/570 nm. JC-1 monomers in the cytoplasm (in both live and apoptotic cells) were detected as a green color at excitation/emission = 490/520 nm.

### 2.12. Caspase-3 Activity Assay

Caspase-3 activity was determined using the fluorescent substrate DEVD-afc according to the manufacturer's instructions (BioVision, Inc., Mountain View, CA, USA). Briefly, at 6, 12, and 24 h both Tan IIA-treated and untreated cells were isolated by scraping the dish with a rubber policeman and centrifuged at 400 ×g for 10 min. The supernatant was removed, the pellet was suspended in 100 *μ*L of lysis buffer (BioVision, Inc.), and incubated at 4°C for 10 min followed by centrifugation at 12.000 ×g for 10 min. Aliquots (50 *μ*L) of the supernatant were removed and placed in a 96-well microplate containing reaction buffer (BioVision, Inc.). Substrate was added and the microplate was incubated at 37°C for 2 h. Activity was monitored by the linear cleavage and release of the afc-side chain and compared with a linear standard curve generated on the same microplate.

### 2.13. Statistical Analyses

All experiments were performed at least three times. Values are expressed as means ± SEM. Statistical analyses were carried out using one-way analysis of variance (ANOVA) followed by the least significant difference (LSD) post hoc *t*-test. Statistical significance was accepted at the level of *P* < 0.05.

## 3. Results

### 3.1. Inhibitory Effect of Tan IIA on the Growth of Keratinocytes

**Figure 2:**
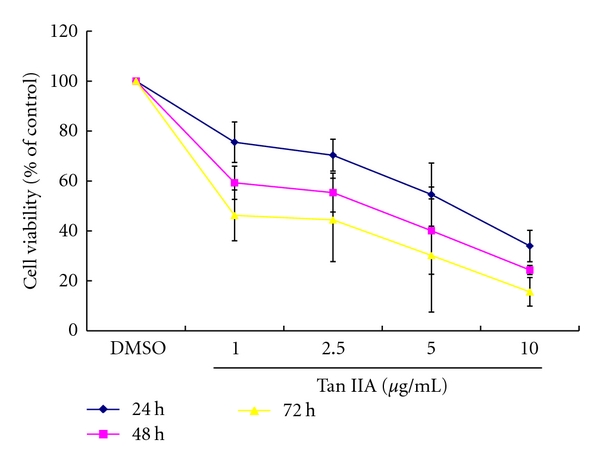
Effects of tanshinone IIA on cell viability of mouse keratinocytes. Dose- and time-dependent growth inhibition was observed at concentrations ranging from 1.0 to 10.0 *μ*g/mL. Results are expressed as a percentage of the control (100%) and represent the mean ± SD of independent experiments performed at least in triplicate.

We investigated the concentration- and time-dependent patterns of the antiproliferation effect of Tan IIA, using the MTS/PMS assay (Figure 2). After exposure to Tan IIA, keratinocytes showed consistent growth retardation compared with the control group. The data unambiguously confirmed that Tan IIA exerted potent growth inhibitory effects on keratinocytes in a concentration- and time-dependent manner. The IC_50_ values for the effects of Tan IIA on the growth of keratinocytes when incubated for 24, 48, and 72 h were 4.33 ± 1.35, 1.89 ± 0.65, and 1.14 ± 0.87 *μ*g/mL, respectively.

### 3.2. Analysis of Clonogenic Survival

**Figure 3:**
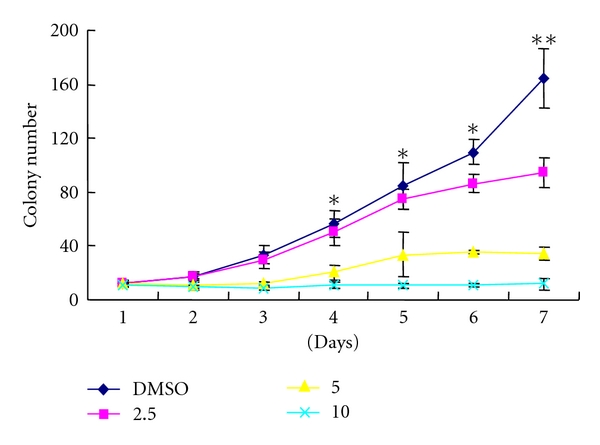
Inhibition of colony formation of mouse keratinocytes by tanshinone IIA. Colony formation inhibition occurred in a dose-dependent manner. Results are means ± SD of independent experiments performed in triplicate. The asterisk indicates a significant difference between control and tanshinone IIA-treated cells (*P < 0.05, **P < 0.01).

To further measure the inhibitory effect of Tan IIA on keratinocytes, colony number growth curves were generated for each clone over a 1-week period (Figure 3). By day 3, the control group produced 33.5 ± 7.1 colonies while Tan IIA-treated groups exhibited only 12.0 ± 1.0 (*P* < 0.05) and 8.25 ± 1.01 (*P* < 0.05) colonies with 2.5 and 5.0 *μ*g/mL, respectively. By day 7, the DMSO control group of keratinocytes produced much higher colony numbers of 164 ± 22 while Tan IIA groups only produced 95.0 ± 11.1 (*P* < 0.05), 34.5 ± 4.9 (*P* < 0.05), and 11.8 ± 3.8 (*P* < 0.05) colonies with 2.5, 5.0, and 10.0 *μ*g/mL Tan IIA, respectively. These results show that with increasing concentrations of Tan IIA, cell colony formation was progressively inhibited. The highest concentration of Tan IIA (10.0 *μ*g/mL) exhibited almost complete inhibition of colony formation.

### 3.3. Tan IIA-Induced Alterations of Cellular Morphology

**Figure 4:**
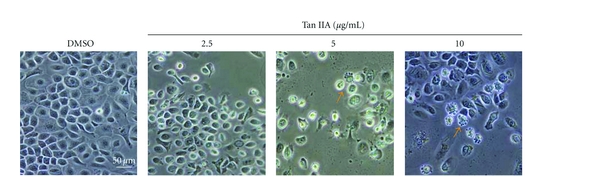
Morphology of keratinocytes exposed to tanshinone IIA. Detection of tanshinone IIA-induced apoptosis in keratinocytes under light microscopy. With increasing concentrations of tanshinone IIA, different features of chromatin clumps (several different forms of chromatin aggregation, arrows) were clearly observed. Detection of apoptotic cells gradually increased in a dose-dependent manner. Marked morphological changes of cellular apoptosis, including condensation of chromatin and nuclear fragmentation, were clearly observed. Each experiment was performed in triplicate. The scale bar in the first panel represents 50 *μ*m for all sections.

After exposure to different concentrations of Tan IIA for 24, 48, or 72 h, marked morphological changes indicative of apoptosis were observed (Figure 4). These included condensation and fragmentation of the nuclear chromatin, shrinkage of the cytoplasm, and loss of membrane asymmetry (arrows). Such apoptotic features were rarely observed in the control group. These morphological changes indicate that Tan IIA has the capability of inducing cellular apoptosis in cultured mouse keratinocytes. We also obtained the same results in human HaCat keratinocytes (data not shown).

### 3.4. Analysis of Apoptosis Induced by Tan IIA

**Figure 5:**
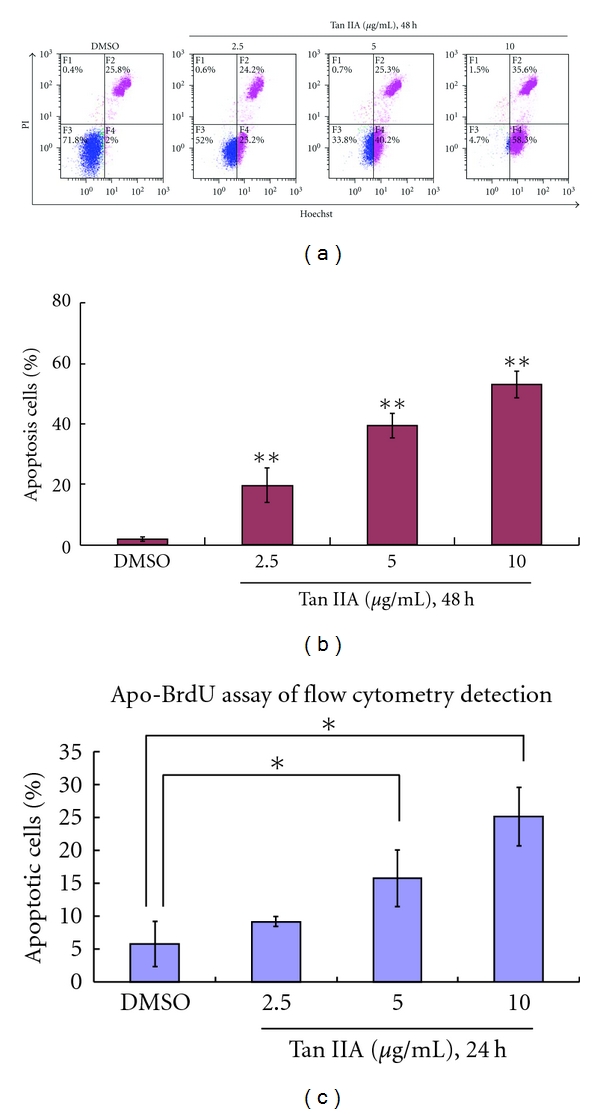
Tanshinone IIA induces apoptosis in mouse keratinocytes. Detection of tanshinone IIA-induced apoptosis and necrosis was measured by (a) flow cytometry after Hoechst 33324/PI staining. (b) Graph represents means ± SD of percent of apoptotic cells of flow cytometry results. (c) Detection of tanshinone IIA-induced apoptosis with Apo-BrdU staining followed by flow cytometry. Asterisk marks indicate a significant difference between control and tanshinone IIA-treated cells (*P < 0.05, **P < 0.01).

To determine the contribution of cell death to Tan IIA-induced reduction in cell growth, we employed two different methods to detect apoptotic cells: Hoechst staining and Apo-BrdU labeling followed by flow cytometry detection. Hoechst staining was observed under fluorescence microscopy, and more frequently showed condensed and fragmented nuclei in Tan IIA-treated groups than in the control group. These findings were further confirmed by flow cytometry detection of both Hoechst and Apo-BrdU labeling methods. As shown in Figure 5(a), with increasing exposure concentrations of Tan IIA, the percentage of apoptotic cells was increased (23.2%, 40.2%, and 58.3%, for 2.5, 5.0 and 10.0 *μ*g/mL Tan IIA, resp.,) as compared to that in the control group, which exhibited only 2% apoptotic cells, representing about a 25-fold increase at the highest concentration of Tan IIA as compared with the control (Figure 5(b)). Similar results were obtained with the Apo-BrdU assay (Figure 5(c)).

### 3.5. Analysis of DNA Laddering

**Figure 6:**
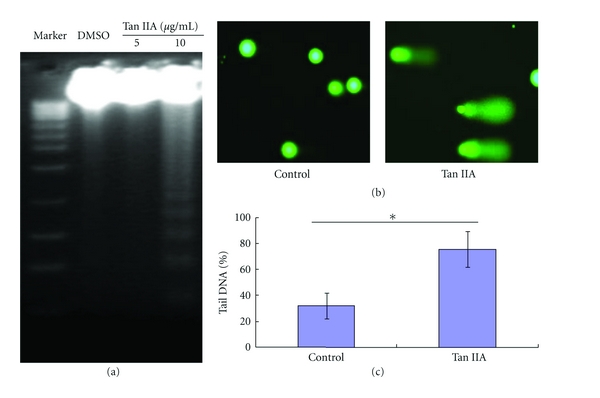
DNA damage analysis by fragmentation assay and OxiSelect Comet assay. (a) DNA ladder was clearly observed at 10 *μ*g/mL concentration of tanshinone IIA. (b) OxiSelect Comet Assay Kit results of tanshinone IIA treatment. Keratinocytes were untreated (left) or treated (right) with tanshinone IIA (10 *μ*g/mL, 24 h) and analysis of apoptosis was performed by the Comet assay. (c) Graph represents means ± SD of percent of tail DNA% from 100 cells.

Detection of DNA laddering on electrophoresis gels was used to confirm the morphological findings regarding the apoptotic action of Tan IIA. As shown in Figure 6(a), Tan IIA-treated cells exhibited DNA fragmentation at a concentration of 10 *μ*g/mL. Single-cell gel electrophoresis revealed that the number of DNA strand breaks was significantly higher in cells of the Tan IIA-treated groups compared with control (Figures 6(b) and 6(c)).

### 3.6. Role of the Caspase Signaling Pathway

**Figure 7:**
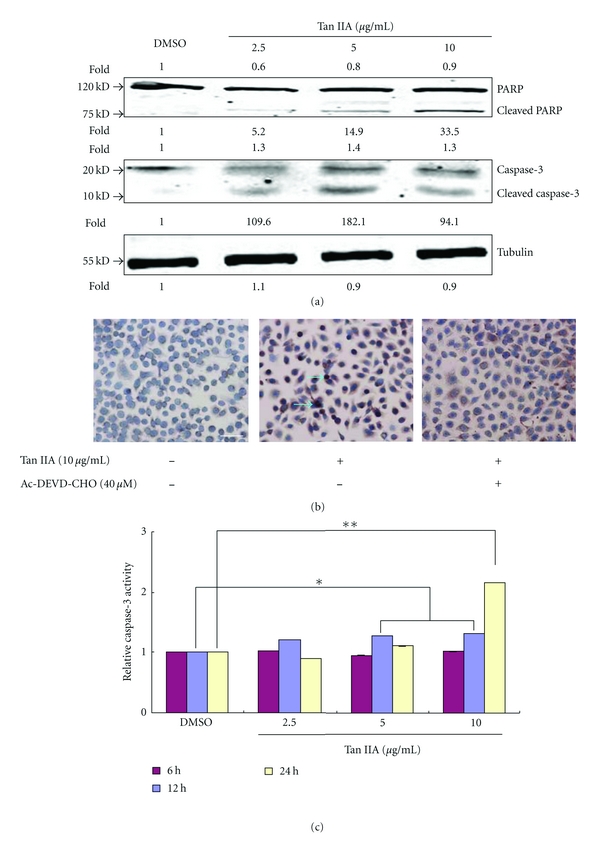
Analysis of apoptosis-related modulators induced by tanshinone IIA. (a) Expression of cleaved caspase-3 and PARP was detected by Western blotting. (b) Keratinocytes were exposed to 10 *μ*g/mL tanshinone IIA for 48 h in the presence (+) or absence (−) of 40 *μ*M Ac-DEVD-CHO (a specific caspase-3 inhibitor). Caspase-3 was detected by immunocytochemistry. (c) Cleaved caspase-3 activity was compared in control versus tanshinone IIA-treated cells using the caspase-3 fluorogenic substrate DEVD-afc. Scale  bar = 50 *μ*m. Asterisk marks indicate a significant difference between control and tanshinone IIA-treated cells (*P < 0.05, **P < 0.01).

**Figure 8:**
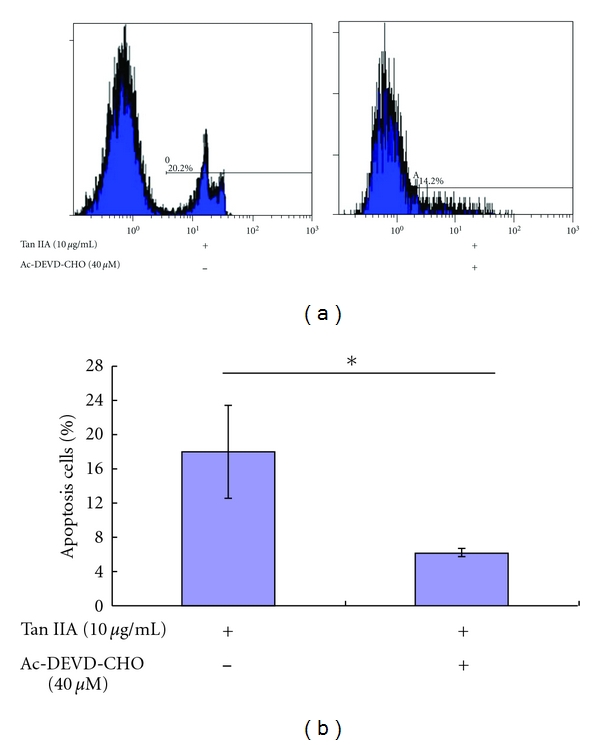
Effects of Ac-DEVD-CHO on tanshinone IIA-induced apoptosis in keratinocytes. Apoptosis was determined by Apo-BrdU staining. Ac-DEVD-CHO attenuated tanshinone IIA-induced apoptosis, indicating that tanshinone IIA causes apoptosis by a caspase-dependent pathway. The asterisk indicates a significant difference between control and tanshinone IIA-treated cells (*P < 0.05).

The activation of caspase proteases is well known in the induction process of apoptosis. Using Western blot analysis, we confirmed that expression of caspase-3 and PARP proteins was altered at different concentrations of Tan IIA exposure, ranging from 2.5 to 10 *μ*g/mL. Tan IIA resulted in a dose-dependent increase in the cleavage of caspase-3 and PARP (Figure 7(a)), which triggered the apoptotic process. We treated cells with Ac-DEVD-CHO, a caspase-3 specific inhibitor, in the presence of Tan IIA, to further confirm whether or not caspase-3 played a direct role in Tan IIA-induced apoptosis. The protein expression of caspase-3 induced by Tan IIA, measured by immunohistochemistry, was decreased (Figure 7(b)). At the same time, we also used flow cytometry to further confirm that Ac-DEVD-CHO attenuated Tan IIA-induced apoptosis in keratinocytes (Figure 8). Apoptotic percentage was decreased from 18.0% to 6.19% (*P* < 0.05). Using an ELISA method, we also determined the expression levels of caspase-3 and caspase-8. The expression of caspase-3 was increased at 12 h and 24 h (*P* < 0.05; Figure 7(c)) while there were no effects on caspase-8 expression (*P* > 0.05; data not shown). These results suggest that the growth inhibitory effect of Tan IIA on keratinocytes occurs through an apoptotic pathway induced by one of the caspase cascades.

### 3.7. Tan IIA Induced Apoptosis of Keratinocytes without Translocation of AIF

**Figure 9:**
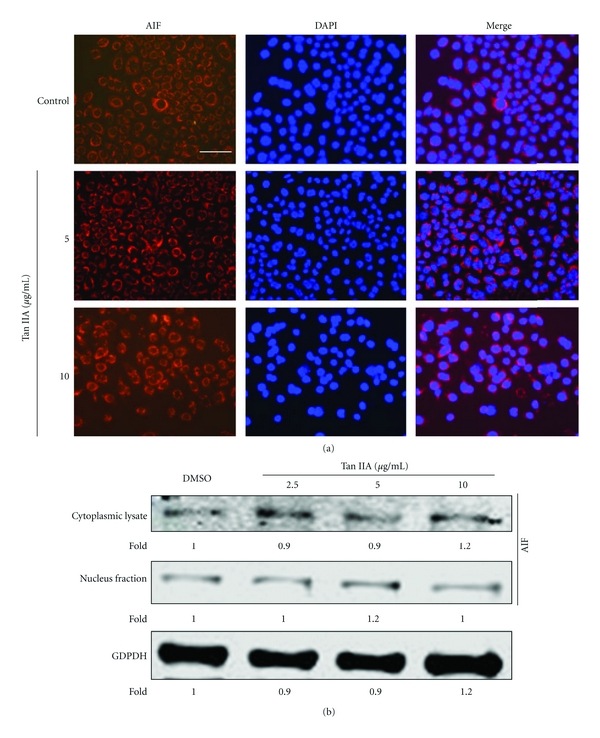
Tanshinone IIA-induced apoptosis is mediated without AIF translocation. (a) Keratinocytes were treated with tanshinone IIA (5 and 10 *μ*g/mL) for 24 h, incubated with antibody against AIF, and Alexa Fluor 488- or 594-conjugated secondary antibodies (Invitrogen) were used for immunofluorescence staining. Nuclei were secondarily stained with DAPI. Purple nuclei due to overlap of AIF (red fluorescence) and nuclear staining (blue fluorescence) means translocation of AIF was not observed. The scale bar in the first panel represents 50 *μ*m for both sections. (b) Western blot analysis showed no difference in AIF expression between the nucleus and cytoplasm.

Apoptosis might proceed through the activation of both caspase-dependent and -independent pathways [16]. AIF, which is localized to the mitochondria and released in response to death stimuli [17], was originally identified as a mitochondrial protein involved in cell death through a caspase-independent pathway [18, 19]. We examined whether or not AIF played a role in Tan IIA-induced apoptotic cell death. AIF was analyzed by observing its release from the mitochondria and translocation to the nucleus by immunofluorescence microscopy. As shown in Figure 9(a), immunofluorescence microscopy showed that AIF did not translocate to the nucleus at exposures to several concentrations of Tan IIA. Western blot analysis also showed that there was no difference in AIF expression between mitochondrial and cytosolic fractions (Figure 9(b)), which is consistent with Tan IIA-induced apoptosis being at least partially caspase dependent.

### 3.8. Tan IIA-Induced Mitochondrial Apoptotic Pathways

**Figure 10:**
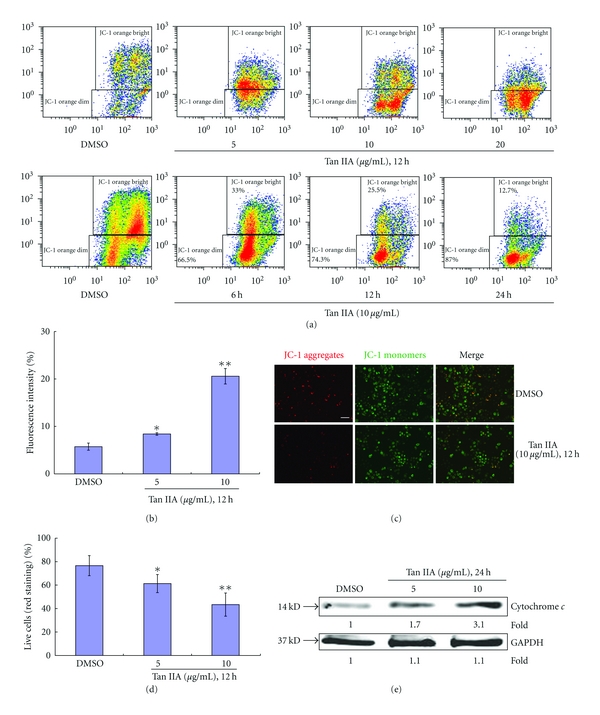
Tanshinone IIA-induced mitochondrial apoptotic pathways. (a) Mitochondrial membrane potential detection assay followed by flow cytometry determination. Tanshinone IIA decreased mitochondrial membrane potential occurred by concentration- and time-dependent patterns. (b) Histograms represent means ± SD of JC-1 orange staining. (c) Cytochrome c was detected as a 14 kD band. GAPDH protein expression was used as the protein loading control. (d) Histogram of representative areas each from the control and treated groups. (e) Mitochondrial red staining (JC-1 aggregates) are a sign of intact, live cells, which is more frequently found in the control group whereas green cytosolic staining (JC-1 monomers) is present in all groups. To quantify the number of cells with intact mitochondrial membrane potential, the number of cells with mitochondrial red staining was counted in 10 separate fields for each group and expressed as a percentage of the total number of cells in the same field. The scale bars represent 50 *μ*m. Asterisk marks indicate a significant difference between control and tanshinone IIA-treated cells (*P < 0.05, **P < 0.01). The scale bar in the first panel of (c) represents 50 *μ*m for both sections.

To further explore the molecular mechanisms of Tan IIA in apoptotic pathways in keratinocytes, we investigated the influence of mitochondrial apoptosis signaling. Flow cytometry and fluorescence microscopy following JC-1 staining revealed a decrease of mitochondrial membrane potential by Tan IIA. As shown in Figures 10(a) and 10(b), the percentage of JC-1 green fluorescence increased in the Tan IIA-treated cells (due to loss of JC-1 aggregates in the mitochondria) from 5.73% to 34.70%, indicating reduced mitochondrial membrane potential, and the number of the cells with intact mitochondrial membrane potential decreased in a Tan IIA concentration-dependent manner (Figure 10(d)). Subsequently, cytochrome c content in the cytoplasm was increased, as measured by Western blot analysis (Figure 10(e)).

### 3.9. Analysis of Cell Cycle Alterations Induced by Tan IIA

**Figure 11:**
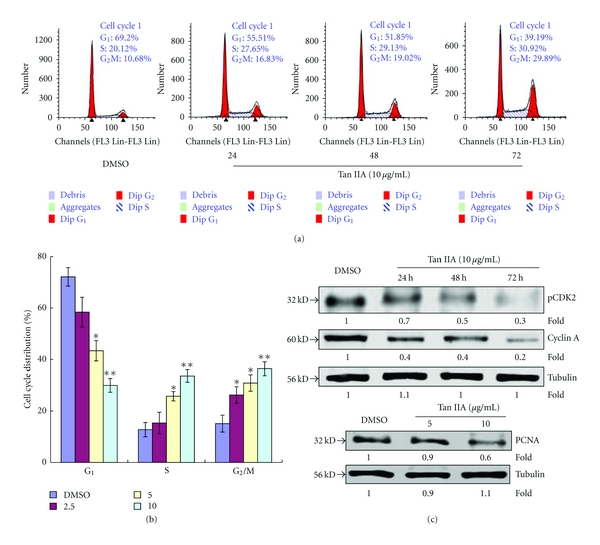
Effect of tanshinone IIA on cell cycle distribution in keratinocytes. (a) Tanshinone IIA treatment resulted in a time-dependent increase in the proportion of cells in the S and G_2_/M phases and a decrease in the proportion of cells in the G_1_ phase. (b) Histograms represent means ± SD of the percent of cell cycle distributions for different concentrations of tanshinone IIA (2.5–10 *μ*g/mL). The asterisk indicates a significant difference between control and tanshinone IIA-treated cells (*P < 0.05). (c) Expression of cyclin A and pCDK2, which are related to S phase of the cell cycle, was found to be decreased, and expression of PCNA, which is related to cell proliferation, was also downregulated compared to the control. Expression of tubulin was used as a protein loading control.

The inhibition of growth in Tan IIA-treated cells may result from cell cycle effects, cell death, or a combination of the two processes. Thus, the cell cycle distribution of Tan IIA-treated and control cells were determined by fluorescence-activated cell sorting analysis of Krishan-stained cells, which gives a measure of DNA content, to explore another related mechanism of growth inhibition for Tan IIA. As shown in Figure 11(a), Tan IIA-treated cells exhibited a lower proportion of cells in the G_0_-G_1_ phase (55.5%, 51.9%, and 39.2% for 24 h, 48 h, and 72 h exposures to 10.0 *μ*g/mL Tan IIA, resp.) as compared with control cells (69.2%). On the other hand, a concomitant increase was observed in the proportion of cells in the S/M phase (27.6%/16.8%, 29.1%/19.0%, and 30.9%/29.9% for 24 h, 48 h, and 72 h exposures to 10.0 *μ*g/mL Tan IIA, resp.) relative to that observed in controls (20.1%/10.7%; Figures 11(a) and 11(b)). Western blot analysis was completed to compare expression levels of various cell cycle-related proteins between the control and Tan IIA-treated cells. Among them, cyclin A and pCDK2, which are related to the S phase, were decreased. Additionally, PCNA, which was originally identified as an antigen that is expressed in the nuclei of cells during the DNA synthesis phase of the cell cycle [20], and is related to cell proliferation, was also decreased compared with the control (Figure 11(c)).

## 4. Discussion

Tan IIA is a major phytochemical of a well-known Chinese herbal medicine called Danshen from the dried root of *Salvia miltiorrhiza radix* [21]. It has been widely used as a herbal remedy in traditional Chinese medicine to treat psoriasis and other related skin diseases. According to the number and quality of clinical trials with botanicals, the best evidence exists for their use in the treatment of inflammatory skin diseases, such as atopic dermatitis and psoriasis. However, more controlled clinical studies are needed to determine the efficacy and risks of plant-derived products in dermatology [22]. Although clinical results are encouraging, knowledge about the pharmacological actions and clinical applications are incomplete.

Psoriasis is one of the most unexplained and persistent skin disorders. It is characterized by abnormal proliferation and differentiation of keratinocytes, such that these cells multiply up to ten-times faster than normal, typically on the knees, elbows, and scalp. The common form, termed “plaque psoriasis vulgaris,” is observed in more than 80% of patients. In China, psoriasis affects 0.123% of the population and there have been more than 3 million cases reported since 1984 [23].

Although other investigators [24] have made tremendous progress, to our knowledge there have been no studies on the antiproliferative effect of Tan IIA in keratinocytes or the underlying mechanism by which this chemical can be used to treat psoriasis. The purpose of this study was to provide evidence of the growth inhibitory effects of Tan IIA in keratinocytes. The MTS/PMS assay showed that Tan IIA strongly inhibited the proliferation of keratinocytes. The inhibition was both dose and time dependent (IC_50_ = 4.33 *μ*g/mL, 24 h). Tan IIA also inhibited the colony growth potential of cells in vitro.

Analysis of cell cycle status with Krishan staining showed that Tan IIA induced cell cycle arrest at the S phase. Morphological changes such as nuclear condensation were also observed, DNA fragmentation was clearly demonstrated by gel electrophoresis, and quantitative analyses by Hoechst/PI staining and Apo-BrdU methods revealed that Tan IIA-induced apoptosis occurred in a dose- and time-dependent manner. Furthermore, appropriate changes in expression of apoptosis-related proteins such as caspase-3, caspase-8, and PARP were examined by Western blot analysis. Taken together, these data suggest that Tan IIA acts by inducing apoptosis of keratinocytes.

Mitochondrial outer membrane permeabilization (MOMP) is often required for activation of the caspase proteases that cause apoptotic cell death. Various intermembrane space (IMS) proteins, such as cytochrome c, promote caspase activation following their mitochondrial release [25]. Activated caspase-3, a downstream cleavage product of the caspase cascade, plays a central role in the execution of apoptotic cell death. We assessed the effects of Tan IIA on the activation of caspase-3 in keratinocytes. ELISA data demonstrated the dose-dependent ability of Tan IIA to increase caspase-3 activation. To further investigate the role of caspase-3 in Tan IIA-induced apoptosis, keratinocytes were exposed to 10 *μ*g/mL Tan IIA for 48 h in the presence or absence of 40 *μ*M Ac-DEVD-CHO, a specific caspase-3 inhibitor. Ac-DEVD-CHO attenuated the induction of apoptosis by Tan IIA, indicating that Tan IIA induces apoptosis by a caspase-dependent mechanism.

AIF, initially characterized as a protein confined within the mitochondrial IMS of healthy cells, is a key protein that can be released from mitochondria and translocated to the nucleus during caspase-independent apoptosis [26]. Immunofluorescence microscopy, however, showed that AIF was not induced by Tan IIA and there was no translocation from mitochondria. These results suggest that activation of the caspase-independent apoptotic pathway mediated via AIF translocation into the nucleus was not involved in Tan IIA-induced apoptosis of keratinocytes.

Furthermore, our investigations found that the molecular mechanism by which Tan IIA inhibits cell growth also involves cell cycle arrest. To further define the function of Tan IIA in cell cycle pathways, we investigated differential expression profiles of various cell cycle regulatory proteins using Western blot analysis and flow cytometry assays. Cyclin-dependent kinase 2 (CDK2), a member of a highly conserved family of protein kinases that regulates the eukaryotic cell cycle [27], interacts with retinoblastoma-like protein 2, retinoblastoma-like protein 1, cyclin A1 [28, 29], cyclin E1 [30], and p21 [29]. Recent research demonstrated that Cdk2 has an unanticipated role in suppressing Myc-induced senescence, suggesting that inhibition of Cdk2 may have therapeutic efficacy in the treatment of cancer [31]. Cyclin A binds to Cdk2 in the S phase and is required for the cell to progress through the S phase. It has long been believed that Cdk2 and its activator cyclin E play essential roles in the progression of the mitotic cell cycle. However, recent studies revealed that neither Cdk2 nor cyclin E is essential *in vivo*. The expression and activity of the second cyclin binding partner of Cdk2, cyclin A, as well as the expression and degradation of the Cdk2 inhibitor p27Kip1 in the absence of Cdk2 [32], also appear to be important. Cell cycle flow cytometry analysis demonstrated that growth of keratinocytes could be inhibited by treatment with Tan IIA and this was due to time- and dose-dependent arrest in S and G_2_/M phases. Cell cycle arrest was accompanied by downregulation of pCdk2 and cyclin A proteins.

Taken together, our study provides novel insight into the molecular mechanisms that underlie the significant antiproliferative effects of Tan IIA in keratinocytes. The mechanism includes inducing S and G_2_/M phase arrest of the cell cycle and triggering apoptosis through the caspase signaling pathway. S and G_2_/M phase arrest may be mediated by downregulation of pCDK2 and cyclin A proteins. Tan IIA-induced apoptosis occurred by mitochondrial disruption, activating caspase-3, but without affecting AIF translocation. The results partially elucidate the mechanism for therapeutic efficacy of *Salvia miltiorrhiza radix* in treatment of psoriasis.

Although the present work was conducted in normal keratinocytes, an important goal for future studies is to assess the efficacy of Tan IIA in psoriatic keratinocytes. Such an efficacy would be based on finding a drug that was selectively cytotoxic to psoriatic cells. Because such cells have a higher rate of growth and proliferation than normal cells, it is likely that they would be more sensitive to the cytotoxic effects of Tan IIA. Hence, one could presumably find a dose of Tan IIA that produced little effect on growth and proliferation of normal keratinocytes but was cytotoxic to psoriatic keratinocytes.

## Abbreviations

Tan IIA: Tanshinone IIA
DMSO: Dimethyl sulfoxide
MTS/PMS: 3-(4, 5-dimethylthiazol-2-yl)-5-(3-carboxymethoxyphenyl)-2-(4-sulfophenyl)-2H-tetrazolium, inner salt/phenazine ethosulfate
PI: Propidium iodide
PARP: Poly ADP-ribose polymerase
Cdk2: Cyclin-dependent kinase 2.
